# Mapping Polysulfides in Sodium–Sulfur Batteries

**DOI:** 10.1021/acsnano.4c16941

**Published:** 2025-02-28

**Authors:** Esther Lilian Gray, Jung-In Lee, Zhuangnan Li, James Moloney, Ziwei Jeffrey Yang, Manish Chhowalla

**Affiliations:** Department of Materials Science and Metallurgy, University of Cambridge, Cambridge CB30FS, U.K.

**Keywords:** sodium−sulfur batteries, sodium polysulfides, tetraethylene glycol dimethyl ether, in situ Raman spectroscopy, ex situ UV–vis spectroscopy, S_3_^•−^ radical species

## Abstract

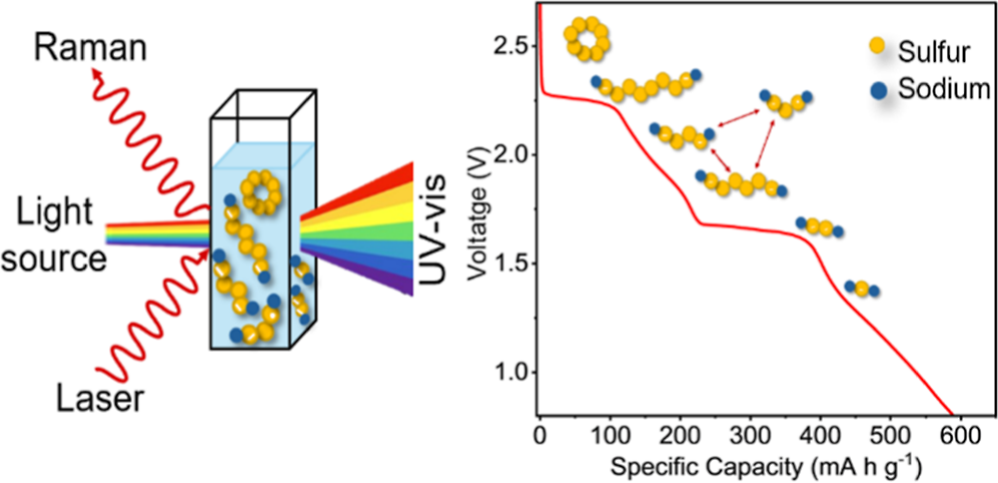

Sodium–sulfur (Na–S) batteries provide lithium-free alternatives to lithium–sulfur (Li–S) batteries. Na–S chemistry has been less studied. Thus, the types of polysulfides (PS) and their evolution during charge–discharge of Na–S batteries are not as well understood as those in the Li–S system. We, therefore, study the formation of different PS in tetraethylene glycol dimethyl ether-based electrolyte during battery operation using *in situ* Raman and *ex situ* ultraviolet–visible (UV–vis) spectroscopies. We start by making reference solutions with different ratios of sodium sulfide (Na_2_S) to sulfur, ranging from pure Na_2_S to Na_2_S:7S, with the sulfur ratio increasing by one integer per solution. We then correlate the UV–vis and Raman peaks to PS species. Our galvanostatic charge–discharge (GCD) and cyclic voltammetry measurements show a total of ten features. Using *ex situ* UV–vis on aliquots and *in situ* Raman spectra from PS solutions at GCD voltage plateaus, we map out sodium polysulfide (NaPS) species at key stages of the charge–discharge cycle. We identify Na_2_S_8_, Na_2_S_4_, and Na_2_S_2_ as intermediates and Na_2_S as the final product. We find that intermediate Na_2_S_6_ forms from disproportionation of Na_2_S_8_ and Na_2_S_4_. We also observe that intermediate PS can also dissociate into S_3_^•–^ radical species, which contributes to loss of active material. Our results provide detailed insights into Na–S chemistry that will be helpful for the development of high performance and stable batteries.

## Introduction

Lithium-ion batteries are currently low in energy density and contain critical metals.^1^ The use of sulfur as a cathode and lithium metal as an anode offers the possibility of achieving high energy density without the need for critical materials in applications requiring high energy density batteries.^2^ Sodium–sulfur (Na–S) batteries are interesting for the same reason as sodium-ion batteries–they offer an alternative to lithium-based batteries, which may have cost advantages in some applications such as large-scale grid energy storage.^3^

However, Na–S batteries face several challenges that are similar to lithium–sulfur (Li–S) batteries. These include polysulfide (PS) shuttling, cyclability, dendrite growth, and the volume expansion of sodium. New separator designs,^4−6^ electrolytes,^7−11^ and cathodes^12−14^ or catholytes^15^ have been reported to address some of these challenges. Studies have also focused on reaction pathways involved in the formation and decomposition of sodium polysulfides (NaPS) during cycling.^11,16^ Most studies use ether-based electrolytes and *ex situ* characterization techniques.^8,17,18^*In situ* analysis has been done for Na–S batteries with carbonate electrolytes or catholytes.^7,15,19,20^ In particular, Xu et al. used *in situ* Raman to monitor the formation of Na_2_S_*x*_ from 2.70 to 1.20 V discharge voltage.^7^ However, further insights into voltages at which various PS form and reduce during cycling could be useful for optimizing the performance of Na–S batteries through an improved electrode or electrolyte design.

Understanding PS reactions may identify critical rate-determining steps and capacity loss mechanisms during cycling. During discharge, elemental sulfur is converted into successively smaller liquid phase NaPS (Na_2_S_*n*_, 4 ≤ *n* ≤ 8) and then finally to solid Na_2_S_2_ and sodium sulfide (Na_2_S).^21^ However, the slow formation kinetics of solid sodium sulfides restrict discharge efficiency and lead to irreversible capacity loss during cycling.^21^ The commonly observed discharge capacity of Na–S batteries (1050 mA h g^–1^) falls between theoretical capacities of forming Na_2_S_2_ and Na_2_S^21^ as the final product. However, it is not clear what the final discharge products are in experimental cells.^21^ Some reports suggest a mixture of Na_2_S_2_ and Na_2_S or Na_2_S_3_ and Na_2_S_2_.^8,21,22^ Previous Na–S studies using a tetraethylene glycol dimethyl ether (TEGDME) electrolyte^8,15,17,18,20,23^ have reported S_6_^2–^ ↔ 2S_3_^•–^ contributes significantly to the loss in active material.

Here, we investigate Na–S batteries with metallic molybdenum disulfide (MoS_2_) nanosheet electrodes as a sulfur host, 1 M sodium trifluoromethanesulfonate (NaCF_3_SO_3_) in TEGDME as the electrolyte, and sodium metal as the anode. The aim is to study PS products using *ex situ* ultraviolet–visible (UV–vis) spectroscopy and *in situ* Raman spectroscopy. Using these methods, we identify Na_2_S_8_, Na_2_S_4_, and Na_2_S_2_ as intermediates and Na_2_S as the final product. Our results also suggest that intermediate Na_2_S_6_ does not form directly from the reduction of sulfur but from disproportionation of Na_2_S_8_ and Na_2_S_4_. Dissociation of Na_2_S_6_ or the disproportionation of Na_2_S_4_ leads to the formation of S_3_^•–^ radical species, which contributes to loss of active material.

## Results and Discussion

### Battery Charge/Discharge

**Figure 1:**
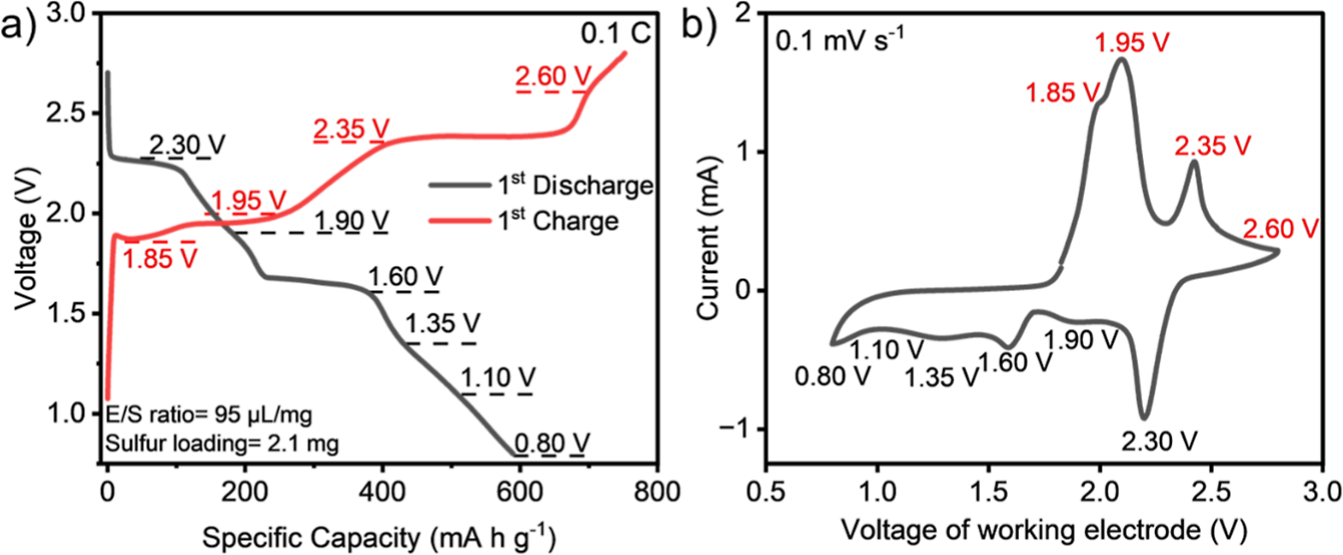
(a) The GCD of 1st cycle at 0.1 C rate achieving initial capacity of 600 mA h per gram (g^–1^) of sulfur and (b) CV plot of Na–S battery with metallic phase MoS_2_ nanosheets as sulfur cathode host and 1.0 M NaCF_3_SO_3_ TEGDME electrolyte. The plateaus in GCD curves correspond to the peaks in the CV curves (red = charge and black = discharge) between a working voltage of 2.6–0.80 V.

A Na–S battery was assembled using sulfur dispersed on metallic MoS_2_ host, sodium anode, and 1 M NaCF_3_SO_3_ TEGDME as the electrolyte (see Experimental Methods for preparation of the cathode and Supporting Information for characterization of material in Figure S1). We have previously reported that the metallic MoS_2_ is a good host for Li–S batteries.^24^ We, therefore, investigated its suitability as a cathode host for the Na–S battery system. The experimental details for coin cell manufacture are described in the Experimental Methods section. The first galvanostatic charge–discharge (GCD) cycle voltage profiles are presented in Figure 1a, along with the corresponding cyclic voltammetry (CV) plot in Figure 1b. The capacity of this coin cell was found to be ∼600 mA h g^–1^ with other cells performing within a standard deviation of ±150 mA h g^–1^ (Refer to Figure S2a for the GCD of the first cycle across five cells). The typical cycling performance of Na–S batteries with metallic MoS_2_ cathode hosts is shown in Supporting Information Figure S2b. The corresponding impedance measurements in Supporting Information Figure S2c show that the resistance of the cell increases with cycling. Several CV plots showing how the plateaus and peaks change with cycling are provided in Supporting Information Figures S2d,e with differential capacity curves of the first cycle in Figure S2f.

Figure 1a shows that during charging, the plateaus occur at 1.85 1.95, 2.35, and 2.60 V, while features occur at 2.30 1.90, and 1.60 V and lower voltages in the discharge curve. The CV plot replicates these plateaus in the form of oxidation and reduction peaks, as shown in Figure 1b. The curves in Figure 1a are similar to previous Na–S battery studies.^8,15,17,18,23^ Typically, ∼2.30 and 1.60 V plateaus are attributed to liquid–liquid conversion reaction of Na_2_S_8_ to Na_2_S_4_. Voltages 1.60–1.10 V correspond to liquid–solid conversion reaction of Na_2_S_2_ and Na_2_S.^11,25^ It has also been noted that the TEGDME solvent encourages the disproportionation of S_4_^2–^ and dissociation of S_6_^2–^ to S_3_^•–^ radical, which causes loss of active material.^23^

### UV–Vis on Reference Solutions of Sodium PS

**Figure 2:**
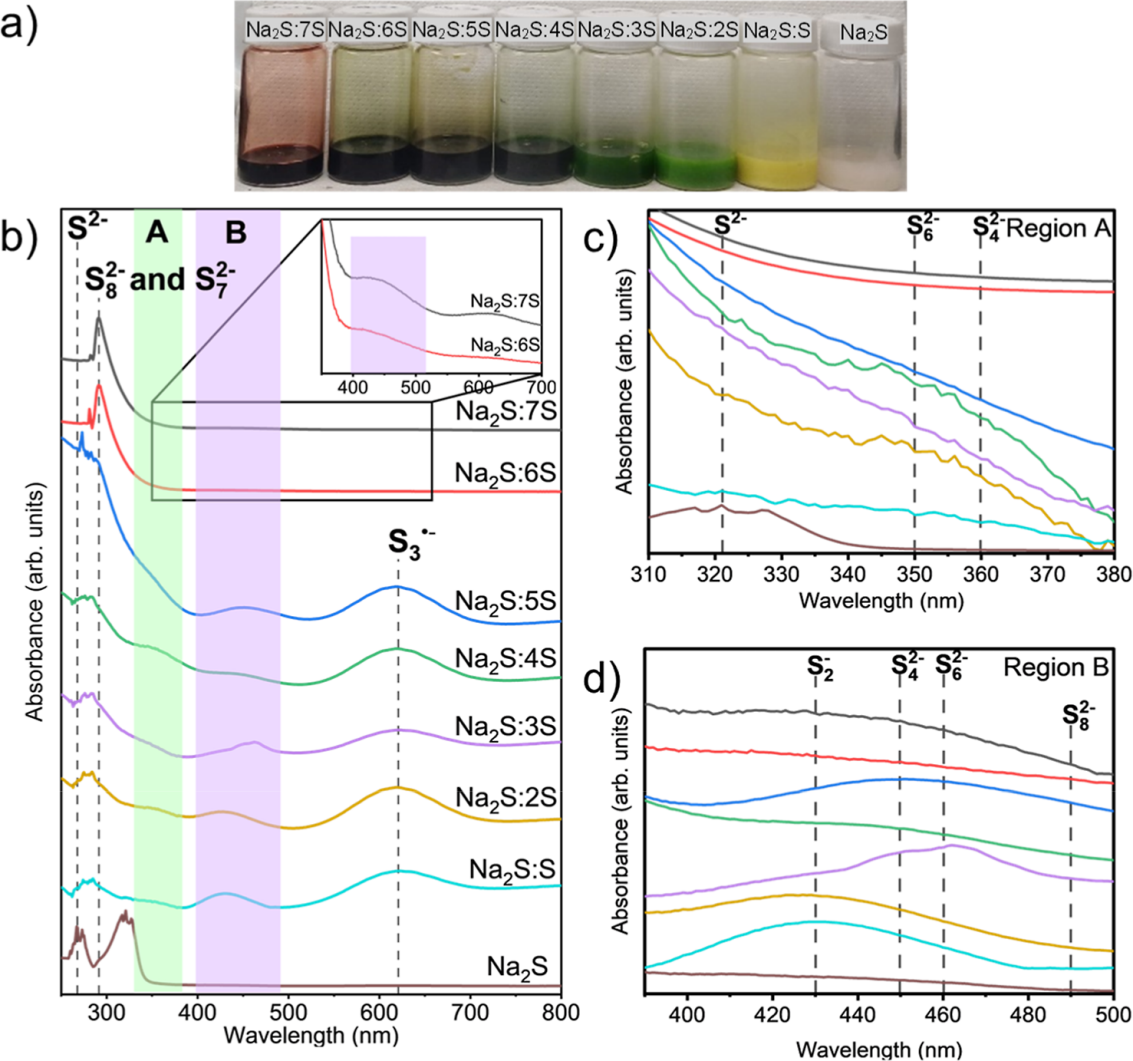
(a) Photographs of 0.2 M NaPS solutions in 1.0 M NaCF_3_SO_3_ and TEGDME. Solutions are labeled with different molar ratios of sulfur to Na_2_S. (b) UV–vis spectra of the reference PS solutions in 1.0 M NaCF_3_SO_3_ in TEGDME. Consistent peaks are labeled individually with regions A and B containing multiple peak shifts. (c) Zoomed in region A and (d) region B, both identifying the individual peaks within the respective regions and the responsible species are summarized in Table 1.

For UV–vis and Raman spectroscopies, 0.2 M solutions of liquid-phase NaPS were prepared in the electrolyte (see Experimental Methods for details). Figure 2a shows photographs of the reference solutions of liquid-phase NaPS with a concentration of 0.2 M Na_2_S_*n*_ with different compositions. These solutions serve as references with predetermined ratios of S and Na_2_S. UV–vis spectra of these reference solutions are shown in Figure 2b (see Figure S3 and Table S1 for detailed UV–vis results). These spectra provide an indication of the peaks corresponding to various PS that form during cycling of a battery.

The various peaks observed in UV–vis spectra (Figure 2b) from the reference liquid-phase PS solutions are labeled in Table 1.^23,26−33^ The spectra show that the concentration of shorter chain PS increases when the sodium-to-sulfur ratio is increased. The results also indicate that different PS species can coexist. Thus, the labels refer to the sodium-to-sulfur ratio of the prepared solutions rather than the absolute concentration of PS species.

**Table 1:** PS Identification from UV–Vis Spectra^a^

wavelength (nm)	sample containing peak/region	polysulfide species
267	Na_2_S	S^2–^
285–290	Na_2_S:7S	S_8_^2–^ and S_7_^2–^
	Na_2_S:6S	
	Na_2_S:5S	
	Na_2_S:4S	
	Na_2_S:3S	
	Na_2_S:2S	
	Na_2_S:S	
region A 320–360	Na_2_S:5S	S^2–^, S_4_^2–^, S_6_^2–^
	Na_2_S:4S	
	Na_2_S:3S	
	Na_2_S:2S	
region B 430–460	Na_2_S:5S	S_2_^–^, S_4_^2–^, S_6_^2–^, S_8_^2–^
	Na_2_S:4S	
	Na_2_S:3S	
	Na_2_S:2S	
	Na_2_S:S	
620	Na_2_S:5S	S_3_^•–^
	Na_2_S:4S	
	Na_2_S:3S	
	Na_2_S:2S	
	Na_2_S:S	

a Three consistent peaks and two distinct regions labelled in Figure 2 were used to identify the PS species.

We observe 3 consistent peaks and 2 distinct regions in the UV–vis spectra. We label the regions as A and B and the 3 consistent peaks are labeled as S^2–^, S_8_^2–^ and S_7_^2–^and S_3_^•–^ for clarity. The first peak at around 267 nm corresponds to the S^2–^ PS.^27^ S_8_^2–^ and S_7_^2–^ are characterized by peaks in the range of 285–290 nm.^28^ These peaks are the strongest for Na_2_S:7S and Na_2_S:6S solutions. The shoulder next to the S_8_ and S_7_^2–^ peak is hypothesized to correspond to S_8_^2–^, S_7_^2–^, or S_6_^2–^ species, as it is consistently observed in all three solutions (Na_2_S:7S, Na_2_S:6S, and Na_2_S:5S). However, further work is necessary to definitively assign this peak. The S_4_^2–^ species show absorption at 360 nm (region A) and 450 nm (region B), while S_6_^2–^ is identified by peaks at 350 nm (region A) and 460 nm (region B).^27,29,34^ The 450 and 460 nm were observed in Na_2_S:3S solution. However, only the 460 nm peak was present in the Na_2_S:3S, Na_2_S:4S, and Na_2_S:5S solutions. Additionally, Na_2_S:S and Na_2_S:2S show a peak at 430 nm which was deduced to be from S_2_^–^ species.^27,30^ Finally, the radical species S_3_^•–^ may be attributed to the absorption peak at 620 nm.^23,26,27,29,31,33^

### Raman on Reference Solutions of Sodium PS

**Figure 3:**
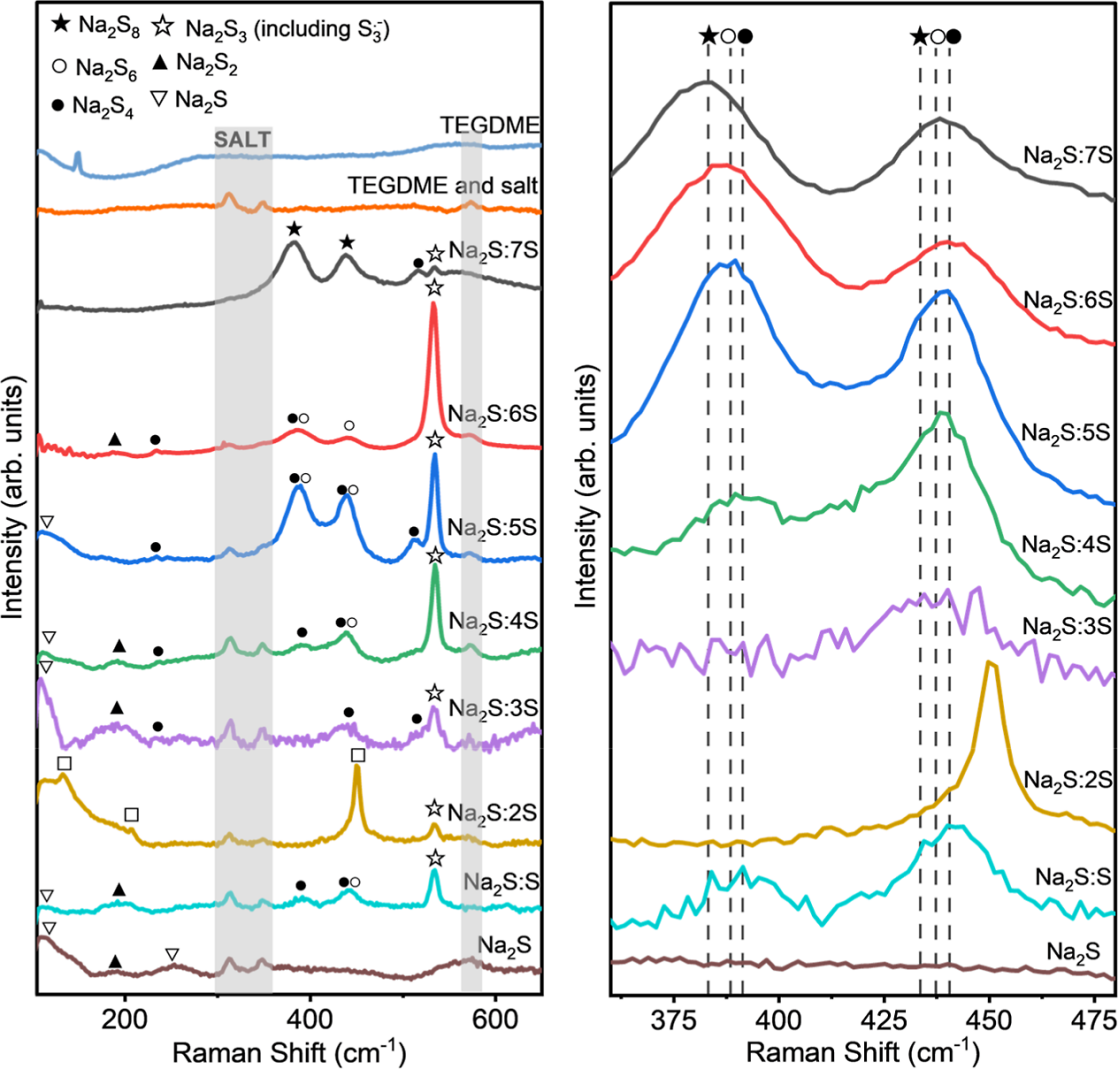
(a) Raman spectra of PS solutions used as references in 1.0 M NaCF_3_SO_3_ and TEGDME with labels identifying each peak with a PS. Vertical shaded regions identify salt and TEGDME peaks. (b) The zoomed in region between 350 and 480 cm^–1^, shows peaks around 380, 386, and 390 cm^–1^ corresponding to S_8_^2–^ and S_7_^2–^, S_6_^2–^, and S_4_^2–^, respectively. Peaks at 436 cm^–1^, 440, and 442 cm^–1^ also correspond to these species, with shifts in peak positions reflecting changes in concentration as indicated by the vertical dashed lines and associated symbols. Figure b also shows that the relative intensity ratios of the two peaks also change with concentration.

Additional identification of NaPS was conducted by Raman spectroscopy. Raman spectra of reference liquid-phase PS solutions are shown in Figure 3. NaPS corresponding to the Raman peaks are summarized in Table 2 (see also Supporting Information Figure S4). The Raman spectra mostly agree with UV–vis results and also show that several PS species coexist in the reference solutions. In contrast to UV–vis, Raman spectroscopy indicated that Na_2_S is present in almost all compositions, which can be attributed to undissolved Na_2_S. Further, the NaPS species are more distinguishable with Raman spectroscopy.

**Table 2:** Wavenumbers of Raman Peaks Associated with Different PSs in Solution (Different Symbols Correspond to Those in Figure 3)

polysulfide (symbol)	Raman shift(s) (cm^–1^)
S^2–^ (▽)	119, 255
S_2_^2–^ (▲)	192
S_3_^2–^ (□)	132, 207, 450
S_4_^2–^ (●)	234, 390, 442, 518
S_6_^2–^ (○)	386, 440
S_8_^2–^ and S_7_^2–^ (★)	380, 436
S_3_^•–^ (symmetric stretching mode) (☆)	534

Specific peaks observed in Raman spectroscopy and shown in Figure 3a are attributed to different NaPS species. The S^2–^ species (▽) exhibit distinct peaks at 119 and 255 cm^–1^.^35−37^ The peak at 192 cm^–1^ is attributed to the S_2_^2–^ species (▲).^38^ The S_3_^2–^ species (□) peaks are observed at 132, 208, and 450 cm^–1^.^39−42^ The S_4_^2–^ species (●) have characteristic peaks at 390 and 518 cm^–1^.^37,39,43,44^ Additionally, the S_6_^2–^ species (○) are identified by peaks at 386 and 440 cm^–1^,^40,41,45^ while the S_8_^2–^ species (★) show peaks at 380 and 436 cm^–1^.^41−43^ The radical species S_3_^•–^ (☆) can be recognized by the symmetric stretching mode at 534 cm^–1^.^37,41,43,44,46−49^ Thus, the Raman peaks provide insight into various NaPS species present in different states (see Supporting Information Table S2).

Additional observations of the S_3_^•–^ radical signal intensity were made across different NaPS solutions. The radical signal is most prominent in Na_2_S:6S, Na_2_S:5S, and Na_2_S:4S, which can be attributed to its formation via the disproportionation of Na_2_S_4_ or the dissociation of Na_2_S_6_.^40,47,50^ These processes are dictated by the Na_2_S:*n*S ratio, governed by thermodynamic equilibrium and stoichiometric constraints, ultimately influencing the predominant polysulfide species in solution.^40,47,50^

### *Ex Situ* UV–Vis Spectroscopy of Solutions at Different Voltage Plateaus

**Figure 4:**
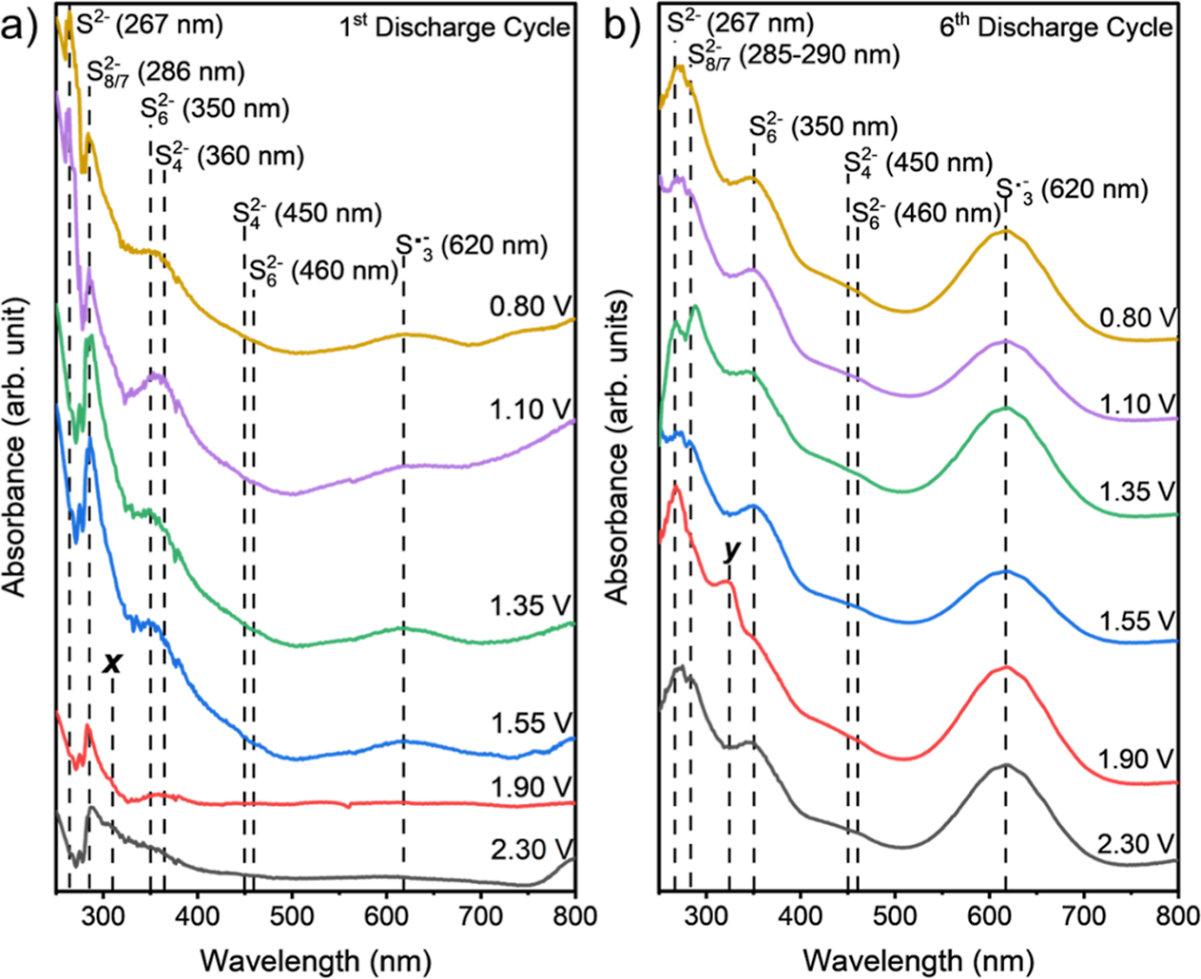
UV–vis spectra of (a) first discharge and (b) sixth discharge of the Na–S battery at 0.1 C rate. The different PSs and their wavelengths are shown by vertically dashed lines.

To understand the evolution of NaPS during battery operation, a Na–S cell was cycled under galvanostatic conditions (0.1 C rate, 1 C = 1672 mA h g^–1^), beginning with a discharge step (see set up in Supporting Information Figure S5). The discharge process was paused at the voltage plateaus indicated in Figure 1a to obtain aliquots of the electrolyte for UV–vis measurements. The UV–vis spectra from the first discharge are shown in Figure 4a. The results were interpreted based on the assigned peaks from reference solutions. The results of the first discharge cycle (Figure 4a) show two peaks at 2.30 V between 285 and 290 nm, which are attributed to the S_8_^2–^ and S_7_^2–^ species. This suggests that between 2.60 and 2.30 V, the conversion of S_8_ to Na_2_S_8_/Na_2_S_7_ occurs. Additionally, a peak at 310 nm (labeled “*x*”) is present, indicating the presence of cyclic S_8_, similar to what has been reported by Han et al.^27^ at 2.30 V. At 1.90 V, a broad peak centered at 360 nm emerges, indicating the formation of S_4_^2–^ species. This peak becomes asymmetrical and sloped from 1.55 V onward, continuing to increase as the battery discharges further. The results suggest the formation of S_6_^2–^ species, while S_4_^2–^ remains, as indicated by an incline at 450 nm and the persistent 360 nm peak. This suggests that both S_6_^2–^ and S_4_^2–^ species increase in concentration within the electrolyte. It is inferred that the PS S_6_^2–^ dissipates in the electrolyte, and it may contribute to battery capacity fading. At 1.55 V, along with the formation of the S_6_^2–^ species, aliquots begin to show a peak at 620 nm, indicating the presence of the S_3_^•–^ radical. From the simultaneous appearance of S_6_^2–^ and the S_3_^•–^ radical, it is inferred that the S_3_^•–^ radical is formed either from the dissociation of Na_2_S_6_ or the disproportionation of Na_2_S_4_.^8,15,17,18,20,23^ There is an observed change in the broadness of the peak at ∼350–360 nm from 1.35 to 1.10 and 0.80 V. At 1.35 V, the peak is asymmetric, suggesting an equilibrium between Na_2_S_6_ and Na_2_S_4_. By 1.10 V, the peak becomes more symmetric, indicating the dominance of Na_2_S_4_. This could potentially result from Na_2_S_6_ dissociating into Na_2_S_3_ at lower potentials. Finally, a peak at 267 nm, attributed to Na_2_S, is evident at 1.10 V.

We also performed the same analysis during the first charge cycle (Supporting Information Figure S6a). We found that the peak associated with S_3_^•–^ radical increases, while the peaks at 267 and 350 nm are observed throughout. This suggests that the S_6_^2–^, S_3_^•–^ radical and Na_2_S PS in the electrolyte are present throughout. However, it should be noted that UV–vis from not all aliquots was measurable because the PS conversion reaction could be concentrated at the surface of the electrode and the aliquots were taken from bulk electrolyte solutions. Consequently, it was not possible to differentiate minor changes in concentration of each reaction in the small volume aliquots compared to measurements on bulk solutions.

We also measured the NaPS concentrations after the sixth discharge and charge cycles (Figure 4b and Supporting Information Figure S6b, respectively) to observe any changes in the concentration of Na_2_S_6_, S_3_^•–^ radical, and Na_2_S species over cycles. In the sixth discharge data, an additional peak at 325 nm (labeled “*y*”) appears at 1.90 V but disappears at lower voltages. This peak has been assigned to the S_3_^2–^ species based on ref (27) and it also appears in the sixth charge data at 1.78 V. Another notable difference is the change in the peak between 330 and 400 nm. In the sixth discharge data, this peak is symmetrical and centered at 350 nm, indicating a strong presence of the S_6_^2–^ species. However, during the charge, the peak transitions from symmetrical to asymmetrical shape, indicating the presence of S_4_^2–^ as the battery charges. This suggests that the S_6_^2–^ species are present in the electrolyte throughout both discharge and charge, while the S_4_^2–^ species are present during discharge until 1.90 V and reappear during charging between 2.30 and 2.60 V.

### *In Situ* Raman Spectroscopy Results

**Figure 5:**
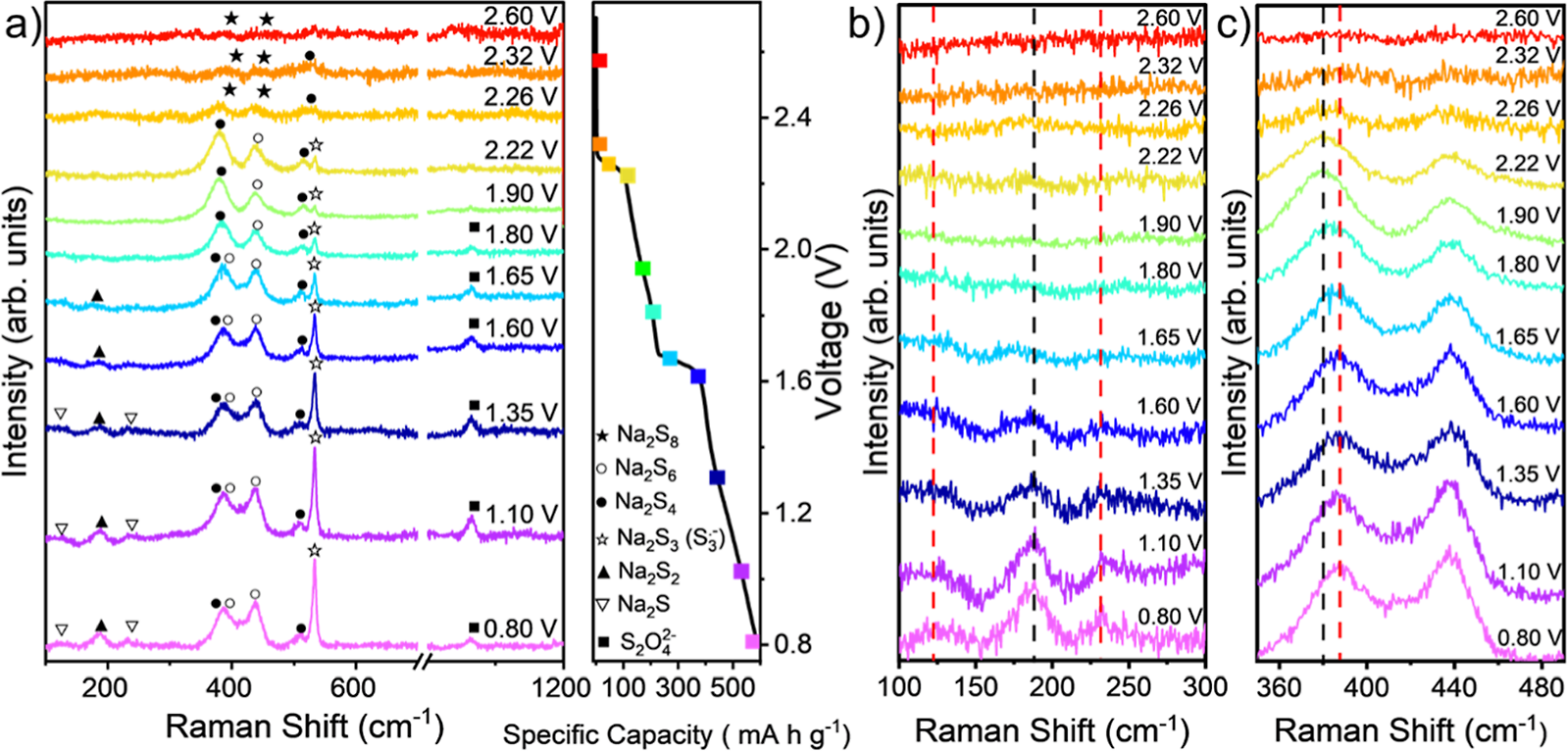
(a) Experimental *in situ* Raman spectra during galvanostatic discharge, presented with specific capacity per gram of sulfur. The spectra are color-coded to correspond to different voltage levels. The Raman cell was run at 0.1 C rate when data were being collected (see Methods). Characteristic peaks are labeled with assigned PS symbols at different voltages as indicated by the plot on the right side. (b) Zoomed in region between 100 and 300 cm^–1^ of the Raman spectra showing the formation of Na_2_S_2_ and Na_2_S. Specifically, presence of Na_2_S_2_ is indicated by the black dashed vertical line. The red vertical lines at 119 and 225 cm^–1^ peaks indicate formation of Na_2_S. (c) Zoomed in region between 350 and 480 cm^–1^ of the Raman spectra showing the peak shift signifying Na_2_S_8_ transforming to Na_2_S_6_ (peak shifting from red dashed line to the black one).

**Figure 6:**
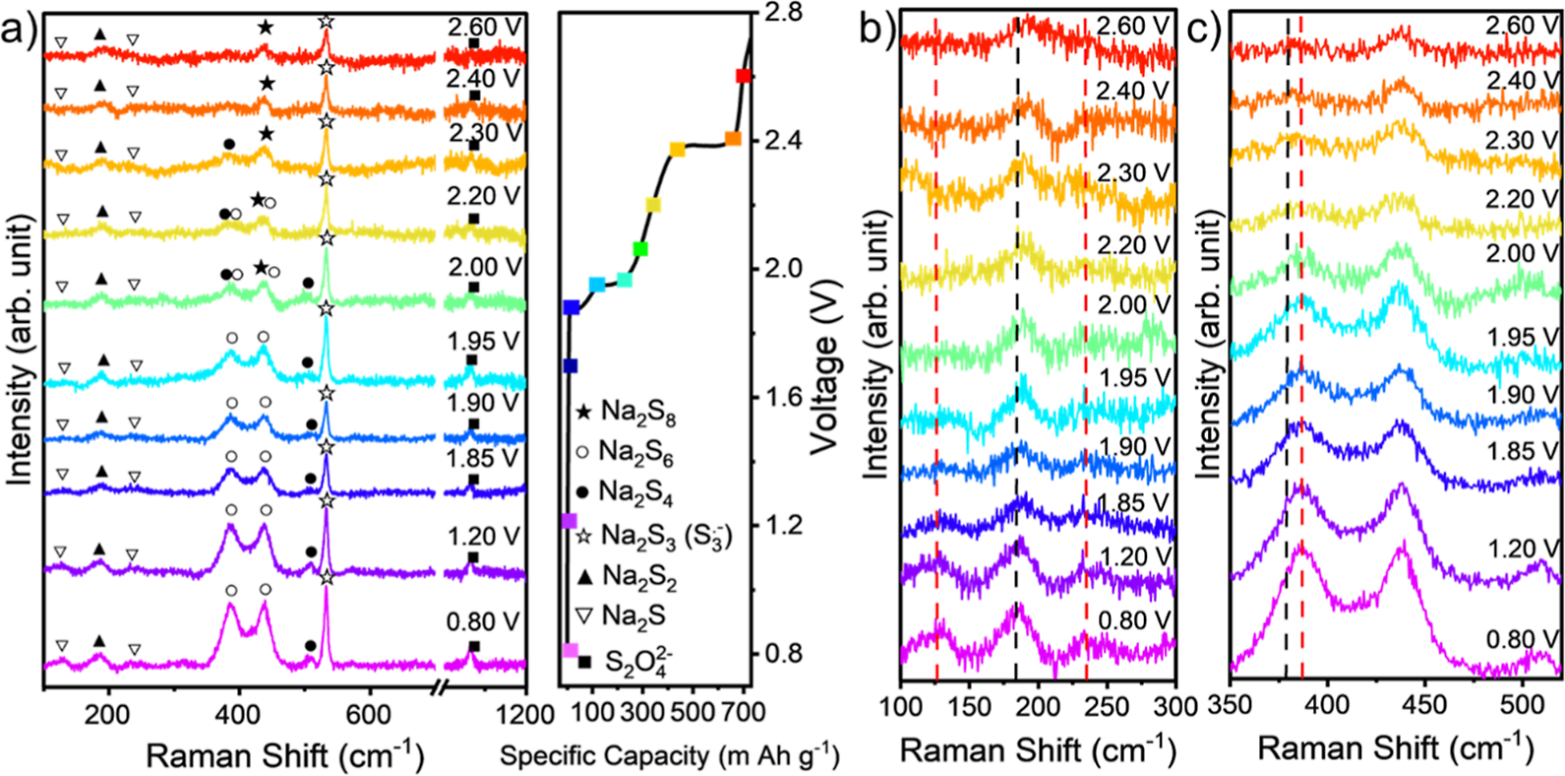
*In situ* Raman measurements during charge cycle. (a) Galvanostatic charge data, presented with specific capacity per gram of sulfur with the corresponding Raman spectra taken at each voltage. Characteristic peaks are labeled with assigned PS symbols at different voltages as indicated in the right figure. (b) Zoomed in region between 100 and 300 cm^–1^ of the Raman spectra. Specifically, the presence of Na_2_S_2_ is indicated by the black dashed vertical line. The red vertical lines at 119 and 225 cm^–1^ peaks indicate formation of Na_2_S. (c) Zoomed in region between 350 and 480 cm^–1^.

To further investigate the reactions within the Na–S battery, *in situ* Raman spectroscopy was conducted (see Experimental Methods). The results for measurements taken during the first discharge and charge cycles are shown in Figures 5 and 6, respectively. The discharge data (Figure 5) show that the 380 and 436 cm^–1^ peaks appear at 2.60 V and remain until 1.80 V. The appearance of the 510 cm^–1^ peak starts at 2.30 V and is present throughout the discharge data. These peaks indicate the presence of Na_2_S_8_ and Na_2_S_4_, respectively, from the reference solutions in Figure 3. From 1.80 V, the peak at 380 cm^–1^ shifts to 386 cm^–1^ which could indicate the beginning of a comproportionation reaction between Na_2_S_8_ and Na_2_S_4_ to form Na_2_S_6_ (as Na_2_S_4_ is observed to be present before Na_2_S_6_ and this corresponds to similar observations within Li–S).^51^ This is seen in Figure 5a and more clearly in its zoomed in regions (Figure 5b,c). In addition, in Figure 5c, the peak at 440 cm^–1^ changes shape becoming more symmetric with the 386 cm^–1^ peak (these changes in peak shifts and shapes could indicate an increase in the presence of Na_2_S_6_). Meanwhile, the peak at 534 cm^–1^ is related to the S_3_^•–^ radical, which first forms at 2.30 V and increases through the discharge cycle. It is also observed that the 192 cm^–1^ peak first appears at 1.65 V, which could be related to the presence of Na_2_S_2_ (as indicated by the black dashed vertical line in Figure 5b). The presence of the 192 cm^–1^ peak corresponds to the second plateau in the GCD curve and remains throughout the discharge step and in the final product. Finally, the 119 and 225 cm^–1^ peaks begin to form at 1.10 V - indicating the formation of Na_2_S.

It is further noted that the intensity of the Na_2_S_4_ peak remains relatively constant after 2.22 V, while Na_2_S_6_ and Na_2_S_3_ peaks grow at lower potentials, even at 0.8 V. This behavior aligns with the sequential polysulfide conversion reactions during discharge, where Na_2_S_6_ and Na_2_S_3_ formation intensifies as the system nears the end of discharge (1.20 V onwards). The persistence of Na_2_S_4_ suggests that it may be in equilibrium with other PS, contributing to the overall conversion mechanism.

The charge data (Figure 6a) shows that between 0.80 and 1.85 V, Na_2_S and Na_2_S_4_ convert back to higher-order PSs from the disappearance of peaks at 119, 225, and 510 cm^–1^. It is also observed that Na_2_S_6_ can be reduced, but it should be noted that the peak at 386 cm^–1^ does not shift back to 380 cm^–1^—suggesting that Na_2_S_6_ dissipates within the electrolyte and does not convert back to Na_2_S_8_. The data also show that the S_3_^•–^ radical is present throughout, indicating that it does not further reduce or convert and stays within the electrolyte. Additionally, Na_2_S_2_ is observed throughout, suggesting that Na_2_S_2_ is not easily reversible and could contribute to capacity decay.

Overall, Raman spectroscopy reveals that Na_2_S_6_ emerges at 2.22 V, a stage where both Na_2_S_8_ and Na_2_S_4_ exist. Notably, Na_2_S_6_ is absent in earlier discharge stages, suggesting it does not form directly from sulfur reduction but instead through disproportionation reactions: Na_2_S_8_ ↔ Na_2_S_6_ + S_2_ and 2Na_2_S_4_ ↔ Na_2_S_6_ + Na_2_S_2_.^40,47,50^ The Raman signal for S_3_^•–^ radicals intensifies during cycling, particularly when Na_2_S_4_ and Na_2_S_6_ are present, indicating the reactions Na_2_S_6_ ↔ S_3_^•–^ + Na_2_S_3_ and 2Na_2_S_4_ ↔ Na_2_S_2_ + 2 S_3_^•–^.^40,47,50^ This is further supported by UV–vis spectroscopy data, which show absorption band shifts consistent with S_3_^•–^ radical formation. The correlation between these spectral changes and the presence of Na_2_S_6_ and Na_2_S_4_ reinforces the role of disproportionation in the reaction mechanism.

### Discharge Conversion Mechanism

**Figure 7:**
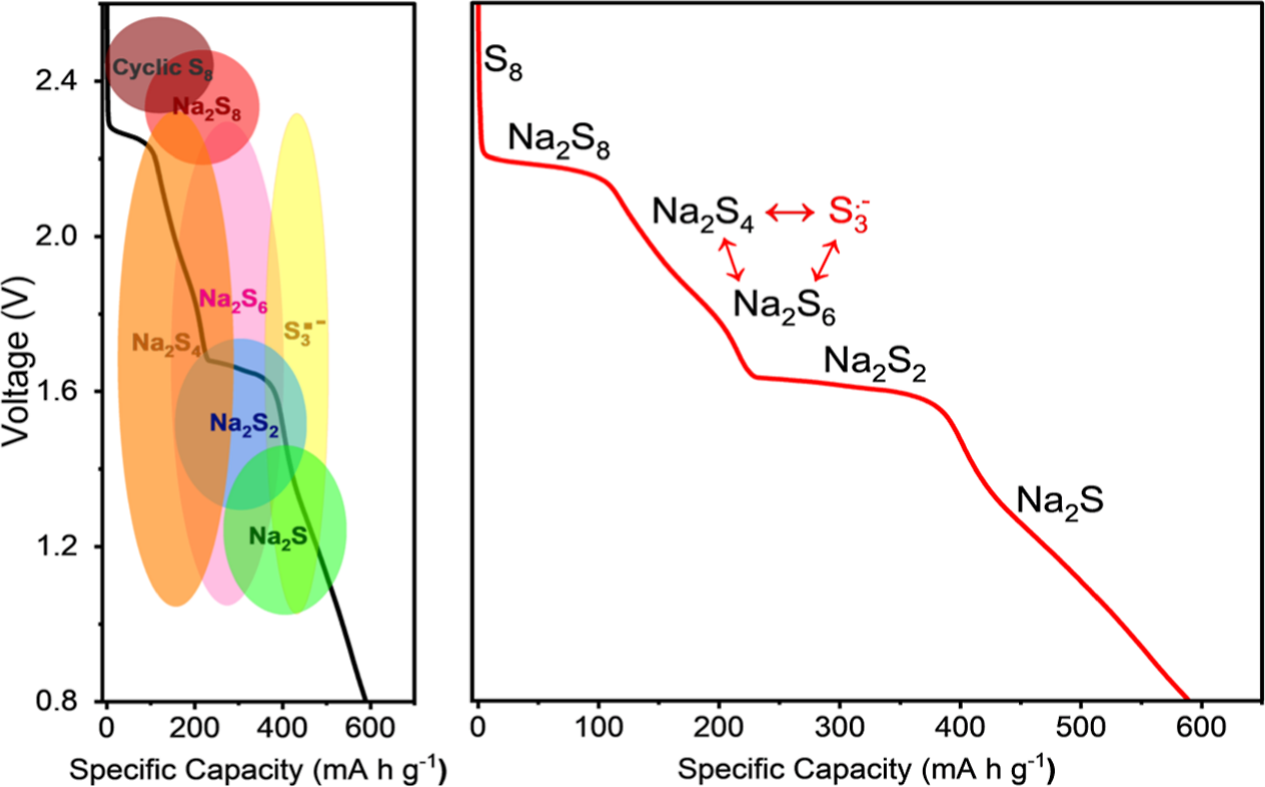
(a) Galvanostatic charge curve of Na–S electrochemical cell showing the different species and their presence (indicated by shaded ellipses and circles) at different voltages. (b) The same charge curve with corresponding pathways for sulfur reduction into different NaPS species with voltage. The stability and presence of various species were surmised from both *ex situ* UV–vis and *in situ* Raman spectroscopies. Indicated in red is the loss of active material side reactions that occur during the sulfur reaction.

We propose the discharge model shown in Figure 7 based on data collected from *ex situ* UV–vis and *in situ* Raman spectroscopies. The model also includes the side reactions that cause loss in active material. Figure 7a shows a range of voltages at which the various NaPS species are found, while Figure 7b shows the specific plateaus and the proposed reaction mechanisms. In particular, we observe a plateau at 2.30 V that is related to the formation of Na_2_S_8_ and Na_2_S_4_, and a second plateau at 1.60 V is associated with the formation of Na_2_S_2_.

## Conclusions

In conclusion, *ex situ* UV–vis and *in situ* Raman spectroscopies were employed to determine the evolution of NaPS species during the charging and discharging of the Na–S cells. Na_2_S_8_, Na_2_S_4_, and Na_2_S_2_ were observed as key intermediates with S_8_ as the starting product and Na_2_S as the final product. Our results suggest that intermediate Na_2_S_6_ does not contribute to the conversion mechanism. However, our results indicate that the formation of Na_2_S_6_ through a comproportionation reaction between Na_2_S_8_ and Na_2_S_4_ contributes to the loss of active material effect when charging. Further, our analysis points to Na_2_S_6_ contributing to the loss in active material by forming S_3_^•–^ radicals through dissociation. The S_3_^•–^ radical can also be formed by the disproportionation of Na_2_S_4_. These processes exist in equilibrium within the electrolyte and typically result in the loss of active material to the electrolyte.

## Experimental Methods

### Preparation of the Reference Sodium Polysulfide Solutions and the Electrolyte

The electrolyte was prepared by dissolving sodium trifluoromethanesulfonate (NaCF_3_SO_3_) (Sigma-Aldrich) in Tetraethylene glycol dimethyl ether (TEGDME) (Sigma-Aldrich) solvent to form a 1 M solution. 0.2 M NaPS solutions were prepared by reacting sodium sulfide (Na_2_S) (Sigma-Aldrich) with sulfur (Nanografi Nano Technology) in stoichiometric proportion in the electrolyte (5 mL). They were prepared and vigorously stirred for 24 h in an argon filled glovebox (<0.1 ppm of O_2_, <0.1 ppm of H_2_O).

### *Ex Situ* UV–vis Measurements

UV–vis spectra for the reference solutions and the *ex situ* study were obtained on a 2-channel spectrometer Lambda 750 (PerkinElmer) using argon-filled, sealed quartz glass cuvettes (Hellma Analytics, QS115). Each aliquot for UV–vis analysis was taken nondestructively and subsequently returned to the system. The electrochemical study for *ex situ* UV–vis spectroscopy was conducted on a SP-50e potentiostat inside an argon filled glovebox. Electrochemical measurements were conducted at 0.1 C rate (1 C = 1672 mA h g^–1^, normalized to the mass of sulfur) in a two-electrode configuration using metallic MoS_2_/sulfur electrodes (12 mm diameter) and 16 mm diameter sodium metal immersed in the electrolyte (30 mL). The aliquots were collected with a mechanical pipet (2 mL) at specified voltages.

### *In Situ* Raman Measurements

The Raman spectra were obtained every 250 s using a Horiba LabRAM Odyssey and analyzed through an ECC-Opto-Std-Aqu, El-Cell with a quartz viewing window. The reference PS were recorded in the same setup without the electrodes.

The electrochemical study for *in situ* Raman spectroscopy was conducted by using a Biologic SP-50e potentiostat. The set up used an airtight electrochemical cell (ECC-Opto-Std-Aqu, El-Cell) with a quartz glass viewing window. Electrochemical measurements were conducted at 0.1 C rate (1 C = 1672 mA h g^–1^, normalized to the mass of sulfur) in a stacked two-electrode configuration. A top-to-bottom stack of metallic MoS_2_/sulfur electrodes (10 mm diameter), glass fiber separator (10 mm diameter), and a sodium metal (12 mm diameter) was created and immersed with electrolyte (∼1 mL).

### Synthesis of Metallic MoS_2_ Nanosheets and Electrodes

Lithiation of 2H-MoS_2_ (0.6 g, <2 μm particle size, Alfa Aesar) was carried out with the *n*-butyllithium in hexane solution (6 mL, 1.6 M, Sigma-Aldrich) and hexane (60 mL, 99% purity).^52^ The solution was stirred at 60 rpm at 80 °C for 72 h under reflux. After cooling the product, it was washed with hexane twice (95% purity, 2 × 50 mL). The product was then dried in a vacuum oven (60 °C, 12 h) and stored in an argon-filled glovebox (<0.1 ppm of O_2_, <0.1 ppm of H_2_O).

### Fabrication of the Cathode

The bulk synthesized product of Li_*x*_MoS_2_ was exfoliated into nanosheets in tetrahydrofuran (0.5 mg/mL) via sonication for 1 h in an ice bath. Then the product was filtered and dried. The cathode was prepared by sonicating 40:60:10 mass ratio of 1T-Li_*x*_MoS_2_, sulfur, and polyvinylidene fluoride in a NMP for 10 min. The resultant solution was stirred for 24 h until the solution was homogeneous. Then, the solution was doctor bladed (150 μm thickness) onto carbon-coated aluminum foil, dried for 24 h at 60 °C under vacuum, and cut into electrodes (12 mm diameter). The volumetric sulfur loading was calculated via the weight of electrodes.

## Electrochemical Characterization

### Assembly and Testing of Coin Cells

The electrochemical performance of the Li_*x*_MoS_2_-based cathodes was evaluated in coin cells (CR2032, Cambridge Energy Solutions), which were assembled in an argon-filled glovebox. The assembly included the components: 12 mm diameter Li_*x*_MoS_2_/sulfur cathode, 16 mm diameter sodium chip, a glass fiber separator, and the electrolyte as prepared previously (flooding the separator with electrolyte). The electrochemical measurements for the coin cells were conducted on a battery cycler (LANDT CT3002A, 1U). GCD tests were performed in the voltage range of 2.6–0.8 V at 0.1 C rate (1 C = 1672 mA h g^–1^, normalized to the mass of sulfur).

## Supporting Information Available

Additional materials characterization; battery analysis; complementary reference database for UV–vis; and Raman data (PDF)
