# Supplementary material for: Mapping Polysulfides in Sodium–Sulfur Batteries
Source: ACS Nano. 2025 Feb 28;19(9):8939–47. doi: 10.1021/acsnano.4c16941 (PMC11912571; doi:10.1021/acsnano.4c16941)
Supplement: Supplementary file 1 — nn4c16941_si_001.pdf [file nn4c16941_si_001.pdf]

**Supplementary Information for**

# Mapping Polysulfides in Sodium-Sulfur Batteries

*Esther Lilian Gray[1], Jung-In Lee[1], Zhuangnan Li[1], James Moloney[1], Ziwei Jeffrey Yang[1], Manish Chhowalla[1*]*

[1]Department of Materials Science and Metallurgy, University of Cambridge, Cambridge, CB30FS, UK

*Corresponding author.

Email: mc209@cam.ac.uk (Manish Chhowalla)

**Characterization of $Li_xMoS_2$**

X-ray diffraction (XRD) and Raman data of 1T-$Li_xMoS_2$ material used for cathodes in Na-S batteries are displayed in Figure S1a and b, showing characteristic peaks of the material as reported previously in our papers including Ref. [1] The material preparation method used here was the same as in our paper Ref. [1] Figure S1c shows that the material was tested in a Na-ion system with 1 M Sodium trifluoromethanesulfonate ($NaCF_3SO_3$) in tetraethylene glycol dimethyl ether (TEGDME) electrolyte, confirming its stability within the Na-S voltage window.

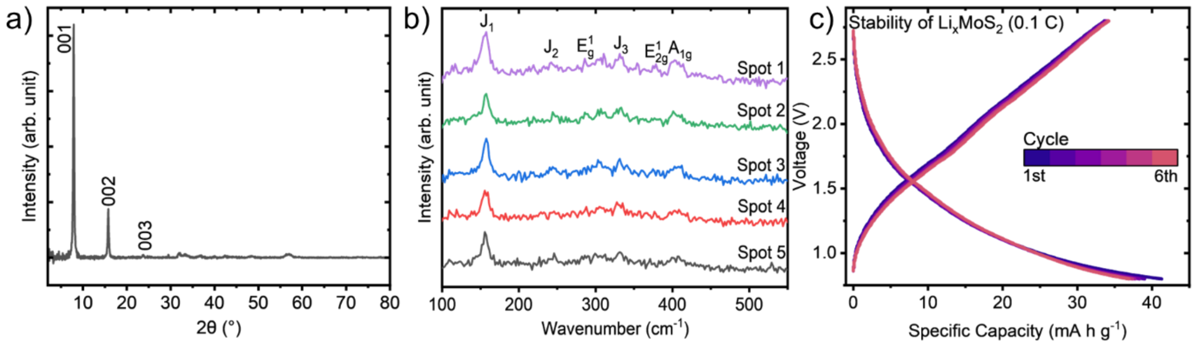


**Figure S1.** a) X-ray diffraction (XRD) b) Raman spectra of 1T-$Li_xMoS_2$ at five random locations and c) the cycling of $Li_xMoS_2$ in Na-ion battery with 1M $NaCF_3SO_3$ TEGDME electrolyte, showing the reaction region.

**Metallic $MoS_2$ nanosheet hosts for Na-S batteries**

We have previously reported that the metallic $MoS_2$ is a good host for Li-S batteries. [1] We therefore investigate their viability for the Na-S battery system. The experimental details for coin cell manufacture are described in the Experimental Methods section. We performed galvanic charge-discharge (GCD) measurements on the coin cells at 0.1 C, the 1$^{st}$ cycle of five cells are shown in Figure S2a and the cycling of one battery for 6 cycles is shown in Figure S2b. It can be seen that the coin cell shows capacity fading from 600 mA h $g^{-1}$ to 200 mA h $g^{-1}$ after six cycles. The improved performance in $MoS_2$ cathode-based battery is due to lower

impedance of the electrodes, shown in Figure S2c, showing that the resistance of the cell increases after 6 cycles. These results indicate that metallic $MoS_2$ nanosheet-based sulfur hosts show promise for Na-S batteries, demonstrating performance comparable to previous studies.[2-5]

Three cyclic voltammetry (CV) scans at 0.1 mV/s scan rate in Figure S2d show that the reduction and oxidation peaks decrease with number of cycles, indicating aging of the battery during discharging and charging and the processes are not reversible due to shuttling of polysulfides. This is also observed in Figure S2e; however, the oxidation peaks reduced from three peaks to two. This may indicate that one of oxidation peaks is not reversible or the higher scan rate does not allow formation of some polysulfides. Finally, the differential capacity curves of the first cycle from Figure S2f, showing that the peaks align with the CV curves.

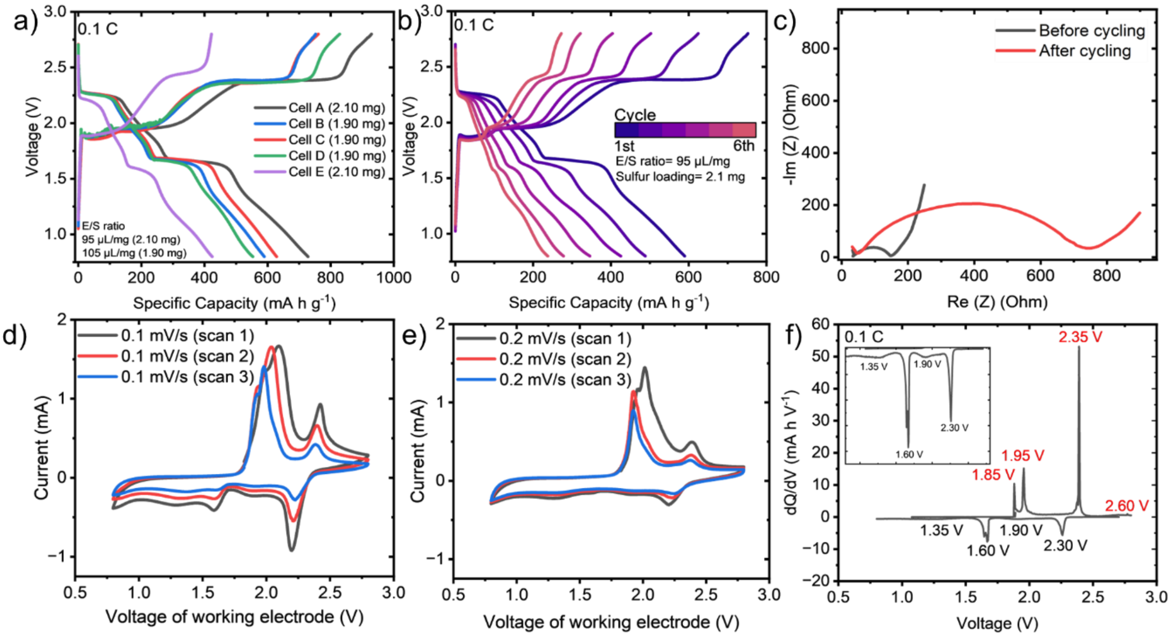


**Figure S2.** a) Galvanic charge-discharge (GCD) graph first cycle across five cells of the Na-S battery with metallic $MoS_2$ nanosheets b) Galvanic charge-discharge (GCD) graph of the Na-S battery with metallic $MoS_2$ nanosheets as the sulfur host, with specific capacity calculated per gram of sulfur. The electrolyte-to-sulfur (E/S) ratio was 95 µL/mg, and the sulfur loading was 2.1 mg. The capacity fading with cycles is attributed to polysulfide shuttling effects. c)

EIS before and after cycling, showing an increase in resistance. CV of Na-S with 1T-$Li_xMoS_2$ as the cathode at different scan rates of d) 0.1 mV/s and e) 0.2 mV/s. Three scans for each are shown. At 0.1 mV/s, three peaks are observed, which at 0.2 mV/s, only two oxidation peaks are present. f) The differential capacity curves of the first cycle from Figure S2b.

**In depth peak assignments for UV-Vis spectra of molar ratio polysulfide solutions**

The UV-Vis spectra displayed in Figure S3 with specific peaks identified in polysulfide solutions with varying molar ratios. These peaks were identified using literature listed in Table S1.

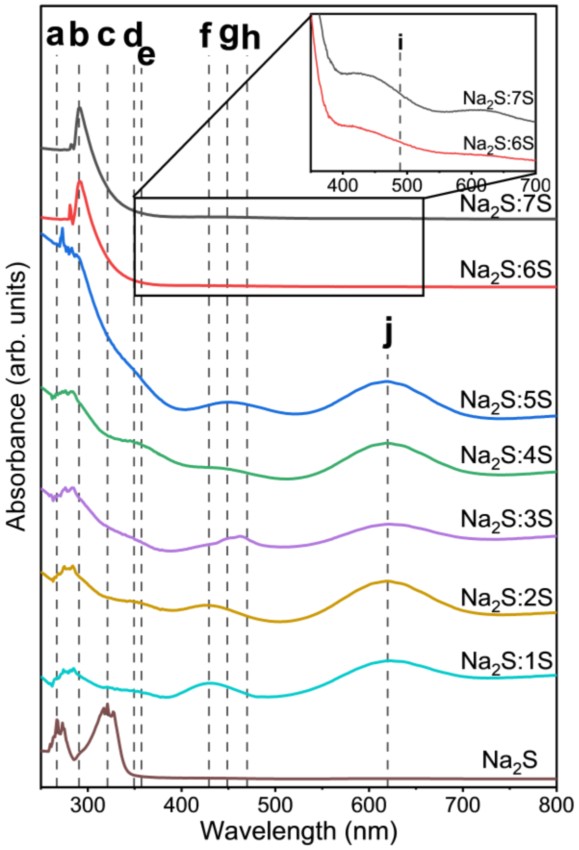


**Figure S3.** UV-Vis spectra of the reference polysulfide solutions in 1.0 M $NaCF_3SO_3$ in TEGDME. Significant peaks are labelled a-j, and the responsible species are identified in Table S1.

**Table S1.** Assignment of polysulfide species peaks from solution of in 1.0 M $NaCF_3SO_3$ and TEGDME with cross reference values from previous Na-S work, lithium polysulfides and our own research.

| Label | Wavelength (nm) | Polysulfide | Reference | Reference reported |
|---|---|---|---|---|
| a | 267 | $S^{2-}$ | [6] | 250 nm for $Li_2S$ in 1 M $LiCF_3SO_3$ DMF |
| b | 285-290 | $S_8^{2-}$ and $S_7^{2-}$ | [7] | 285 nm for $Na_2S_8$ , $K_2S_8$ or $Li_2S_8$ in THF |
| c | 320 | $S^{2-}$ | This work only. | |
| d | 350 | $S_6^{2-}$ | [6, 8, 9] | [6] 340 nm for $Li_2S_6$ in 1 M $LiCF_3SO_3$ DMF<br>[8] 360 nm for $Na_2S_6$ in DMF<br>[9] 350 nm for for $Na_2S_6$ in [$C_4$mim][DCA] |
| e | 360 | $S_4^{2-}$ | [6, 8] | [6] 320 and 340 nm for $Na_2S_4$ in DMF<br>[8] 350 nm $Na_2S_4$ in DMSO |
| f | 430 | $S_2^-$ | [4, 10, 11] | [4] 420 nm for $Li_2S$ in 1 M $LiCF_3SO_3$ DMF<br>[10] $S_2^-$ 400 to 450 nm ($Li_2S_2$ in Hexamethylphosphoramide)<br>[11] 400 nm for $S_2^-$ alkali halide single crystals in KCl |

| | | | | |
|---|---|---|---|---|
| g | 450 | $S_4^{2-}$ | [9, 12, 13] | [9] 440 nm for $Na_2S_4$ in [$C_4$mim][DCA]<br>[12] Reports a broad peak between 400–500 nm region assigning $S_4^{2-}$ and $S_6^{2-}$ species in 1.5 M $NaClO_4$ and 0.3 M $NaNO_3$ in TEGDME<br>[13] 435 nm for $Na_2S_4$ in DMF and DMSO |
| h | 460 | $S_6^{2-}$ | [6, 8, 9, 12] | [6] 450 nm for $Li_2S_6$ in 1 M $LiCF_3SO_3$ DMF<br>[8] 450 nm for $Na_2S_6$ in DMF<br>[9] 460 nm for $Na_2S_6$ in [$C_4$mim][DCA]<br>[12] Reports a broad peak between 400–500 nm region assigning $S_4^{2-}$ and $S_6^{2-}$ species in 1.5 M $NaClO_4$ and 0.3 M $NaNO_3$ in TEGDME |
| i | 491 | $S_8^{2-}$ | [6, 14] | [6] 490 nm for $Li_2S_8$ in1 M $LiCF_3SO_3$ DMF<br>[14] 492 nm for $Na_2S_8$ in DMSO |
| j | 620 | $S_3^{\bullet-}$ | [6, 9, 12, 14, 15] | [6] 600 nm for $S_3^{\bullet-}$ species in 1 M $LiCF_3SO_3$ DMF |

[9] 620 nm for $S_3^{\bullet-}$ species in [C4mim][DCA]

[12] 620 nm for $S_3^{\bullet-}$ species in 1.5 M $NaClO_4$ and 0.3 M $NaNO_3$ in TEGDME

[14] 618 nm for $S_3^{\bullet-}$ species in DMSO

[15] 610 nm for $S_3^{\bullet-}$ species in 1 M $NaPF_6$ Glyme Ether Solvents

**Expanded Raman spectra of sodium polysulfide solutions**

The Raman spectra of sodium polysulfide solutions in the 100–1400 $cm^{-1}$ range, shown in Figure S4, provide an expanded view compared to the spectral range analysed in the main text. This extended wavenumber scale captures peaks up to 1400 $cm^{-1}$. Notably, beyond 600 $cm^{-1}$, the spectra primarily reveal peaks associated with electrolyte and $S_2O_4^{2-}$ species, a product of interactions between the solvent and polysulfides. The spectra are annotated to identify the solvent peaks (highlighted in grey) and salt peaks (marked with black dotted lines). The spectra indicate that polysulfide peaks do not occur above 600 $cm^{-1}$. These peaks were identified using literature listed in Table S2.

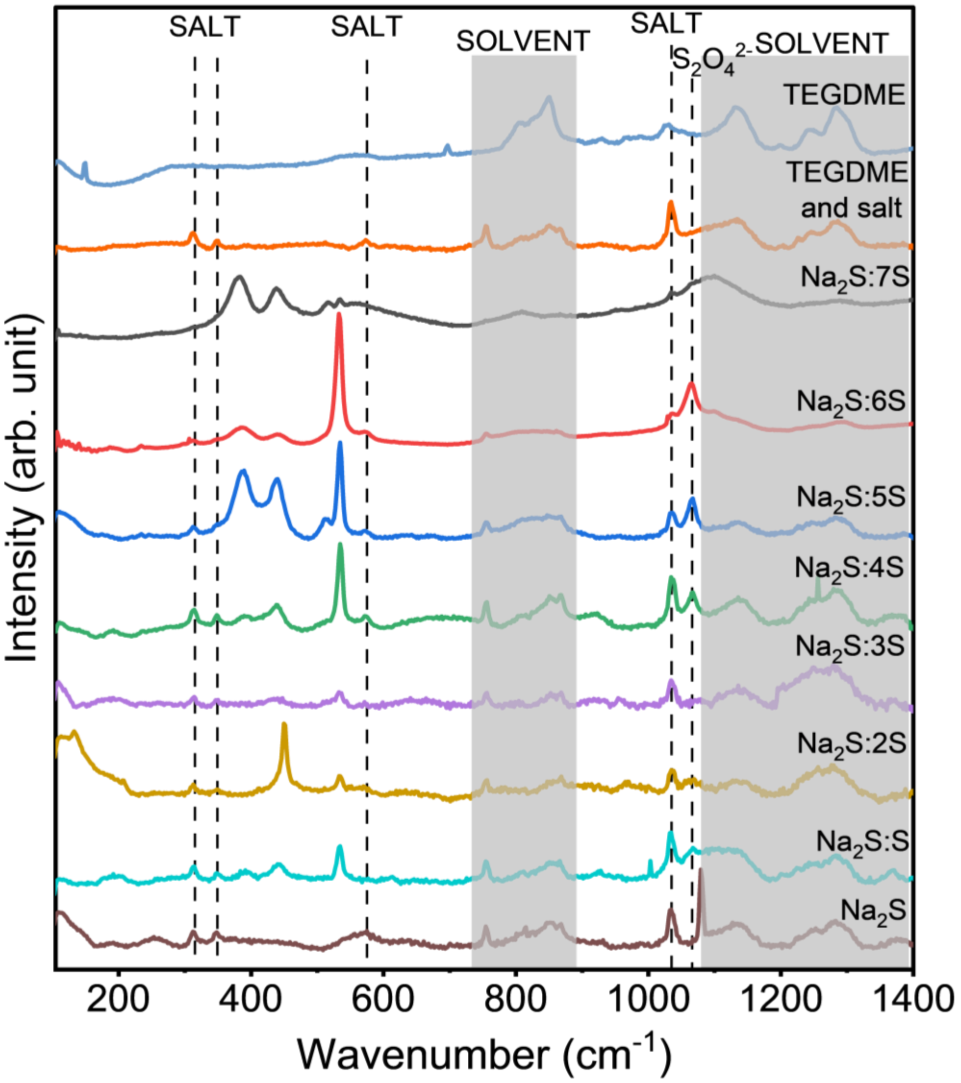


**Figure S4.** Raman spectra of sodium polysulfide solutions in the 100–1400 cm$^{-1}$ range of the sodium polysulfide solutions. Solvent and salt peaks are labelled.

**Table S2.** Polysulfide species observed in this work with solutions of 1.0 M $NaCF_3SO_3$ and TEGDME. Comparison with literature is provided.

| Polysulfide (symbol) | Wavenumber (cm$^{-1}$) | Relevant literature | Reference reported |
|---|---|---|---|
| $S^{2-}$ (▽) | 119 | [16] | [16] reported the 115 cm$^{-1}$ Na-S stretching mode |

| | | | |
|---|---|---|---|
| $S_3^{2-}$ (□) | 132 | [17] | [17] reported 189 $cm^{-1}$ $(Li_2)S_3^{2-}$ in THF (DFT calculation) Within this experimental work this peak is only observed in the molar solution $Na_2S$ + 2S. |
| $S_2^{2-}$ (▲) | 192 | [18] | [18] reported 134.5 $cm^{-1}$ for polycrystalline $Na_2S_2$ assigning as Lib motion of the two $S_2^{2-}$ groups in opposite direction. |
| $S_3^{2-}$ (□) | 207 | [17] | [17] reported 189 $cm^{-1}$ $(Li_2)S_3^{2-}$ in THF (DFT calculation) Within this experimental work this peak is only observed in the molar solution $Na_2S$ + 2S. |
| $S_4^{2-}$ (Bending mode) (●) | 234 | [18-20] | [18] reported 239 $cm^{-1}$ (asymmetric bond) and 206 $cm^{-1}$ (symmetric bond) for polycrystalline $Na_2S_4$ |

| | | | |
|---|---|---|---|
| | | | [19] reported 234 cm$^{-1}$ for $(Li_2)S_4^{2-}$ (bending mode) in 1 M LiTFSI and TEGDME [20] reported 240-245 cm$^{-1}$ assigned to $(Na_2)S_4^{2-}$ in amines |
| $S^{2-}$ (▽) | 255 | [21, 22] | [21] reported 188 cm$^{-1}$ and 210 cm$^{-1}$ as $S^{2-}$. This peak was observed in this experimental work only in the solution $Na_2S$ [22] reported 190 cm$^{-1}$ for $(Na_2)S^{2-}$ in TEGDME |
| $S_{8/7}^{2-}$ (★) | 380 | [17, 23] | [17] reported a DFT calculation assigning 362 cm$^{-1}$ for $(Li_2)S_8^{2-}$ in THF [23] reported 390 cm$^{-1}$ most closely corresponds to $(Li_2)$ $S_7^{2-}$ and $S_8^{2-}$ in 1 M LiTFSI, 0.2 M $LiNO_3$ DME:DOL (1:1) |

| | | | |
|---|---|---|---|
| $S_6^{2-}$ (○) | 386 | [23, 24] | [23] reported 365 $cm^{-1}$ corresponds to ($Li_2$) $S_6^{2-}$ in 1 M LiTFSI, 0.2 M $LiNO_3$ DME:DOL (1:1). [24] reported $K_2S_6$ ($S_6^{2-}$ stretching) at 373 $cm^{-1}$ |
| $S_4^{2-}$ (●) | 390 | [20, 22, 25] | [20] reported 393-400 $cm^{-1}$ assigned to ($Na_2$)$S_4^{2-}$ in amines [22] reported 375 $cm^{-1}$ to ($Na_2$)$S_4^{2-}$ in TEGDME [25] reported 400 $cm^{-1}$ for liquid $Na_2S_4$ |
| $S_8^{2-}$ (★) | 436 | [17, 20] | [17] reported DFT calculation of 427 and 429 $cm^{-1}$ for ($Li_2$) $S_8^{2-}$ in THF [20] reported 414, 424 and 440 $cm^{-1}$ assigned to ($Na_2$)$S_8^{n-}$ in amines |
| $S_6^{2-}$ (○) | 440 | [23, 26] | [23] reported 437 $cm^{-1}$ ($Li_2$)$S_6^{2-}$ in 1 M LiTFSI, 0.2 M $LiNO_3$ DME:DOL (1:1). [26] reports $S_6^{2-}$ at 453 $cm^{-1}$ |

| | | | |
|---|---|---|---|
| $S_3^{2-}$ (□) | 450 | [23, 25, 26] | [23] reported 450 $cm^{-1}$ most closely corresponding to $(Li_2)S_4^{2-}$, $S_3^{2-}$, and $S_4^{-}$ in 1 M LiTFSI, 0.2 M $LiNO_3$ DME:DOL (1:1).<br>[25] reported 440 $cm^{-1}$ to 462 $cm^{-1}$ assigned to the S-S-S of $(Na_2)S_3^{2-}$ anions<br>[26] reports $(Na_2)S_3^{2-}$ at 458 $cm^{-1}$ |
| $S_4^{2-}$ (●) | 518 | [19, 22] | [19] reported 518 $cm^{-1}$ for $S_4^{-}$ in 1 M LiTFSI and TEGDME<br>[22] reports ~500 $cm^{-1}$ to $(Na_2)S_4^{2-}$ in TEGDME |
| $S_3^{\bullet-}$ (Symmetric stretching mode) (☆) | 534 | [19, 20, 22, 23, 27-30] | [19] reported 534 $cm^{-1}$ for $S_3^{\bullet-}$ (Symmetric stretching mode) in 1 M LiTFSI and TEGDME<br>[20] reported 533 to 535 $cm^{-1}$ assigned to $(Na_2)S_3^{\bullet-}$ in amines<br>[22] reports 534 $cm^{-1}$ to $S_3^{\bullet-}$ in TEGDME |

[23] reported 533 $cm^{-1}$ corresponding to ($Li_2$) $S_3^{\bullet -}$ in 1 M LiTFSI, 0.2 M $LiNO_3$ DME:DOL (1:1).

[27] reported 535 $cm^{-1}$ as ($Li_2$) $S_3^{\bullet -}$ in ammonia

[28] and [30] reported 534 $cm^{-1}$ for $S_3^{\bullet -}$ in NaCl

[29] reported 540 cm-1 for an S–S stretch of $a_1$ symmetry of $S_3^{\bullet -}$.

**UV-Vis Electrochemical set up**

The electrochemical set-up for the *ex situ* UV-Vis study for NaPS.

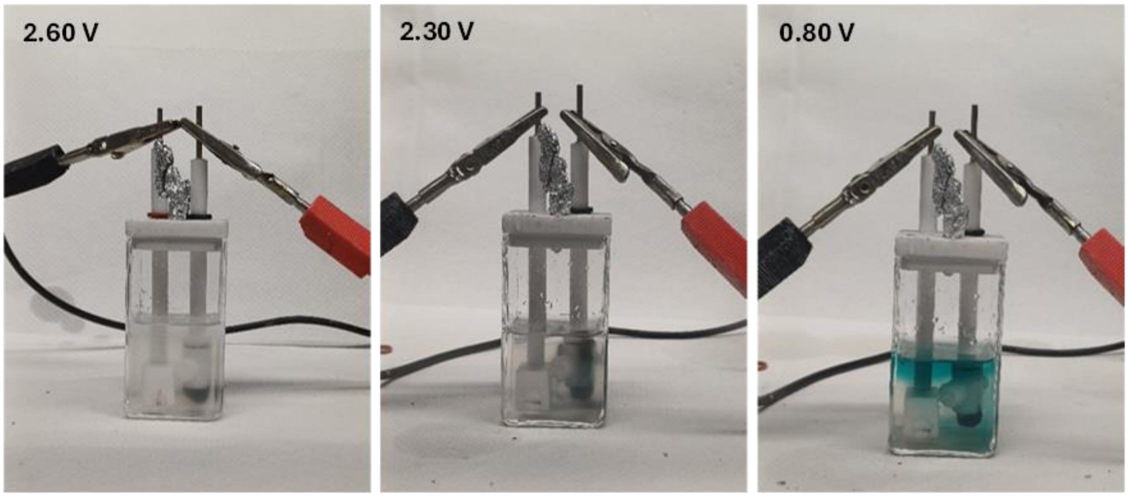


**Figure S5.** The set up of the electrochemical cell for the *ex situ* UV-Vis analysis. The photographs are taken at 2.60 V, 2.30 V and 0.8 V (from left to right) during the first discharge in the first cycle.

UV-vis spectra taken on 1st charge and 6th charge for Na-S *ex situ* is shown in Figure S6. These spectra aimed to reveal how polysulfide species evolve with cycling; however, the data lacked the expected distinction between polysulfide states. This was due to the localized nature of polysulfide conversion, which primarily occurs at the electrode surface, while the aliquots were drawn from bulk electrolyte solutions. As a result, the spectra did not effectively capture minor concentration changes of reaction intermediates, limiting the detection of subtle transformations in these small-volume samples compared to bulk measurements.

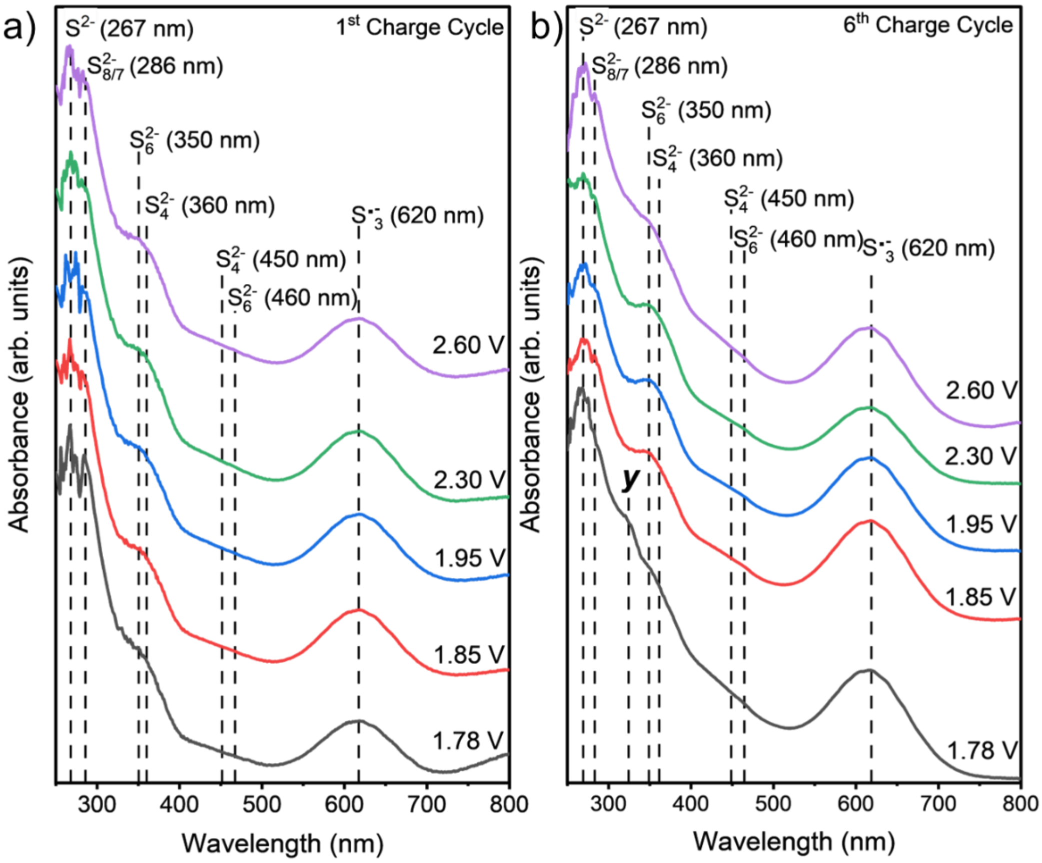


**Figure S6.** UV-Vis spectra of a) 1st charge b) 6th charge cycle of the Na-S battery.

## References


(1) Li, Z.; Sami, I.; Yang, J.; Li, J.; Kumar, R. V.; Chhowalla, M. Lithiated metallic molybdenum disulfide nanosheets for high-performance lithium–sulfur batteries. *Nature Energy* **2023**.

(2) Ryu, H.; Kim, T.; Kim, K.; Ahn, J.-H.; Nam, T.; Wang, G.; Ahn, H.-J. Discharge reaction mechanism of room-temperature sodium–sulfur battery with tetra ethylene glycol dimethyl ether liquid electrolyte. *Journal of Power Sources* **2011**, *196* (11), 5186-5190.

(3) Kim, I.; Park, J. Y.; Kim, C. H.; Park, J. W.; Ahn, J. P.; Ahn, J. H.; Kim, K. W.; Ahn, H. J. A room temperature Na/S battery using a β″ alumina solid electrolyte separator, tetraethylene glycol dimethyl ether electrolyte, and a S/C composite cathode. *Journal of Power Sources* **2016**, *301*, 332-337, Article. DOI: 10.1016/j.jpowsour.2015.09.120 Scopus.

(4) Kohl, M.; Borrmann, F.; Althues, H.; Kaskel, S. Hard Carbon Anodes and Novel Electrolytes for Long-Cycle-Life Room Temperature Sodium-Sulfur Full Cell Batteries. *Advanced Energy Materials* **2016**, *6* (6), 1502185. DOI: https://doi.org/10.1002/aenm.201502185 (acccessed 2025/01/24).

(5) Kim, I.; Park, J.-Y.; Kim, C.; Park, J.-W.; Ahn, J.-P.; Ahn, J.-H.; Kim, K.-W.; Ahn, H.-J. Sodium polysulfides during charge/discharge of the room-temperature Na/S battery using TEGDME electrolyte. *Journal of The Electrochemical Society* **2016**, *163* (5), A611.

(6) Han, D.-H.; Kim, B.-S.; Choi, S.-J.; Jung, Y.; Kwak, J.; Park, S.-M. Time-Resolved In Situ Spectroelectrochemical Study on Reduction of Sulfur in N , N ′ -Dimethylformamide. *Journal of The Electrochemical Society* **2004**, *151* (9), E283.

(7) Tobishima, S.-I.; Yamamoto, H.; Matsuda, M. Study on the reduction species of sulfur by alkali metals in nonaqueous solvents. *Electrochimica Acta* **1997**, *42* (6), 1019-1029.

(8) Kim, B. S.; Park, S. M. In Situ Spectroelectrochemical Studies on the Reduction of Sulfur in Dimethyl Sulfoxide Solutions. *Journal of The Electrochemical Society* **1993**, *140* (1), 115.

(9) Manan, N. S. A.; Aldous, L.; Alias, Y.; Murray, P.; Yellowlees, L. J.; Lagunas, M. C.; Hardacre, C. Electrochemistry of Sulfur and Polysulfides in Ionic Liquids. *The Journal of Physical Chemistry B* **2011**, *115* (47), 13873-13879.

(10) Ledé, B.; Demortier, A.; Gobeltz-Hautecœur, N.; Lelieur, J. P.; Picquenard, E.; Duhayon, C. Observation of the ν3 Raman band of S3− inserted into sodalite cages. *Journal of Raman Spectroscopy* **2007**, *38* (11), 1461-1468.

(11) Holzer, W; Murphy, W. F.; Bernstein, H. J. Raman spectra of negative molecular ions doped in alkali halide crystals. *Journal of Molecular Spectroscopy*, **1969**, 32, 13-23.

(12) Yu, X.; Manthiram, A. Highly Reversible Room-Temperature Sulfur/Long-Chain Sodium Polysulfide Batteries. *The Journal of Physical Chemistry Letters* **2014**, *5* (11), 1943-1947.

(13) Bonnaterre, R.; Cauquis, G. Spectrophotometric study of the electrochemical reduction of sulphur in organic media. *Journal of the Chemical Society, Chemical* Communications 1972, (5), 293-294.

(14) Martin, R. P.; Doub, W. H., Jr.; Roberts, J. L., Jr.; Sawyer, D. T. Electrochemical reduction of sulfur in aprotic solvents. Inorganic Chemistry 1973, 12 (8), 1921-1925.

(15) Carter, R.; NewRingeisen, A.; Reed, D.; Atkinson, R. W., III; Mukherjee, P. P.; Love, C. T. Optical Microscopy Reveals the Ambient Sodium–Sulfur Discharge Mechanism. *ACS Sustainable Chemistry & Engineering* **2021**, *9* (1), 92-100.

(16) Wen, Q.; Wu, Y.; Wang, X.; Zhuang, Z.; Yu, Y. Researches on preparation and properties of sodium polysulphide as gold leaching agent. *Hydrometallurgy* **2017**, *171*, 77-85.

(17) Hagen, M.; Schiffels, P.; Hammer, M.; Dörfler, S.; Tübke, J.; Hoffmann, M. J.; Althues, H.; Kaskel, S. In-Situ Raman Investigation of Polysulfide Formation in Li-S Cells. *Journal of The Electrochemical Society* **2013**, *160* (8), A1205.

(18) Janz, G. J.; Downey, J. R.; Roduner, E.; Wasilczyk, G. J.; Coutts, J. W.; Eluard, A. Raman studies of sulfur-containing anions in inorganic polysulfides. Sodium polysulfides. Inorganic Chemistry 1976, 15 (8), 1759-1763.

(19) Wu, H.-L.; Huff, L. A.; Gewirth, A. A. In Situ Raman Spectroscopy of Sulfur Speciation in Lithium–Sulfur Batteries. *ACS Applied Materials & Interfaces* **2015**, *7* (3), 1709-1719.

(20) Daly, F. P.; Brown, C. W. Raman spectra of sodium tetrasulfide in primary amines. Evidence for sulfide (S42-and S8n-) ions in rhombic sulfur-amine solutions. *The Journal of Physical Chemistry* **1975**, *79* (4), 350-354.

(21) Wang, Y.; Luo, Z.; Liu, D.; Li, Y. Immobilization of mercury in tailings originating from the historical artisanal and small-scale gold mining using sodium polysulfide. *Environmental Science and Pollution Research* **2022**, *29*, 1-17.

(22) Kandhasamy, S.; Nikiforidis, G.; Jongerden, G.; Jongerden, F.; Sanden, M. C. M.; Tsampas, M. Operational strategies to improve the performance and long-term cyclability of intermediate temperature sodium-sulfur (IT-NaS) battery. ChemElectroChem 2021, 8.

(23) McBrayer, J. D.; Beechem, T. E.; Perdue, B. R.; Apblett, C. A.; Garzon, F. H. Polysulfide Speciation in the Bulk Electrolyte of a Lithium Sulfur Battery. *Journal of The Electrochemical Society* **2018**, *165* (5), A876.

(24) Janz, G. J.; Coutts, J. W.; Downey, J. R.; Roduner, E. Raman studies of sulfur-containing anions in inorganic polysulfides. Potassium polysulfides. *Inorganic Chemistry* **1976**, *15* (8), 1755-1759.

(25) El Jaroudi, O.; Picquenard, E.; Gobeltz, N.; Demortier, A.; Corset, J. Raman Spectroscopy Study of the Reaction between Sodium Sulfide or Disulfide and Sulfur:

Identity of the Species Formed in Solid and Liquid Phases. *Inorganic Chemistry* **1999**, *38* (12), 2917-2923.

(26) Chivers, T.; Lau, C. Raman spectroscopic identification of the S4N-and S3-ions in blue solutions of sulfur in liquid ammonia. *Inorganic Chemistry* **1982**, *21* (1), 453-455.

(27) Dubois, P.; Lelieur, J. P.; Lepoutre, G. Identification and characterization of lithium polysulfides in solution in liquid ammonia. *Inorganic Chemistry* **1988**, *27* (1), 73-80.

(28) Chivers, T. Ubiquitous trisulphur radical ion S3−·. *Nature* **1974**, *252* (5478), 32-33.

(29) Chivers, T.; Elder, P. J. W. Ubiquitous trisulfur radical anion: fundamentals and applications in materials science, electrochemistry, analytical chemistry and geochemistry. *Chemical Society Reviews* **2013**, *42* (14), 5996-6005.

(30) Holzer, W.; Murphy, W. F.; Bernstein, H. J. Resonance raman effect of S3− doped in a NaCl single crystal. *Chemical Physics Letters* **1970**, *4* (10), 641-642.
